# The Intrinsic and Extrinsic Implications of RANKL/RANK Signaling in Osteosarcoma: From Tumor Initiation to Lung Metastases

**DOI:** 10.3390/cancers10110398

**Published:** 2018-10-24

**Authors:** Benjamin Navet, Kosei Ando, Jorge William Vargas-Franco, Régis Brion, Jérome Amiaud, Kanji Mori, Hideo Yagita, Christopher G. Mueller, Franck Verrecchia, Clotilde Dumars, Marie-Françoise Heymann, Dominique Heymann, Frédéric Lézot

**Affiliations:** 1 INSERM, UMR 1238, Faculté de Médecine, Université de Nantes, F-44035 Nantes, France; Benjamin.navet@univ-nantes.fr (B.N.); kosei@belle.shiga-med.ac.jp (K.A.); jorge.vargas@udea.edu.co (J.W.V.-F.); regis.brion@univ-nantes.fr (R.B.); jerome.amiaud@univ-nantes.fr (J.A.); franck.verrecchia@univ-nantes.fr (F.V.); clotilde.dumarsbarsi@ch-bretagne-atlantique.fr (C.D.); 2 Department of Orthopedic Surgery, Shiga University of Medical Science, Tsukinowa-cho, Seta, Otsu, Shiga 520-2192, Japan; kanchi@belle.shiga-med.ac.jp; 3 Department of Basic Studies, Faculty of Odontology, University of Antioquia, Medellin AA 1226, Colombia; 4 Centre Hospitalier Universitaire, Hôtel Dieu, F-44035 Nantes, France; 5 Department of Immunology, Juntendo University School of Medicine, Tokyo 113-8421, Japan; hyagita@med.juntendo.ac.jp; 6 CNRS, UPR 9021, Institut de Biologie Moléculaire et Cellulaire (IBMC), Laboratoire Immunologie et Chimie Thérapeutiques, Université de Strasbourg, F-67084 Strasbourg, France; c.mueller@ibmc-cnrs.unistra.fr; 7 INSERM, LEA Sarcoma Research Unit, Department of Oncology and Human Metabolism, Medical School, University of Sheffield, Sheffield S10 2RX, UK; marie-francoise.heymann@ico.unicancer.fr; 8 INSERM, UMR 1232, LabCT, Université de Nantes, Institut de Cancérologie de l’Ouest, site René Gauducheau, F-44805 Saint-Herblain, France

**Keywords:** RANKL/RANK, osteosarcoma, metastases, bone, T-lymphocyte

## Abstract

*Background:* Osteosarcoma is the most frequent form of malignant pediatric bone tumor. Despite the current therapeutic arsenal, patient life-expectancy remains low if metastases are detected at the time of diagnosis, justifying research into better knowledge at all stages of osteosarcoma ontogenesis and identification of new therapeutic targets. Receptor Activator of Nuclear factor κB (RANK)expression has been reported in osteosarcoma cells, raising the question of Receptor Activator of Nuclear factor κB Ligand (RANKL)/RANK signaling implications in these tumor cells (intrinsic), in addition to previously reported implications through osteoclast activation in the tumor microenvironment (extrinsic). *Methods:* Based on in vitro and in vivo experimentations using human and mouse osteosarcoma cell lines, the consequences on the main cellular processes of RANK expression in osteosarcoma cells were analyzed. *Results:* The results revealed that RANK expression had no impact on cell proliferation and tumor growth, but stimulated cellular differentiation and, in an immune-compromised environment, increased the number of lung metastases. The analysis of RANKL, RANK and osteoprotegerin (OPG) expressions in biopsies of a cohort of patients revealed that while RANK expression in osteosarcoma cells was not significantly different between patients with or without metastases at the time of diagnosis, the OPG/RANK ratio decreased significantly. *Conclusion:* Altogether, these results are in favor of RANKL-RANK signaling inhibition as an adjuvant for the treatment of osteosarcoma.

## 1. Introduction

Osteosarcoma is the most common malignant pediatric primary bone tumor. It usually occurs in the metaphysis of the long bones in the second decade of life [1]. The five-year survival rate of patients with localized osteosarcoma reaches approximately 65% and declines to around 20% when lung metastases are detected at the time of diagnosis [2,3,4]. The current conventional therapies for high-grade and metastatic osteosarcoma have not been fully established and have not changed much in recent decades [2,3,4,5,6,7]. Characterization of new therapeutic targets is thus needed, particularly to treat metastatic patients.

Bone tissue is perpetually renewed and bone homeostasis is finely regulated by a balance between bone apposition, carried out by osteoblasts, and bone resorption, for which osteoclasts are responsible [8]. The Receptor Activator of Nuclear factor κB Ligand (RANKL) and its receptors RANK and osteoprotegerin (OPG) are part of the tumor necrosis factor (TNF) and TNF receptor families, respectively TNFSF11, TNFRSF11A and TNFRSF11B. They are all key regulators of bone metabolism [9,10,11]. RANKL, which is mainly secreted by osteoblasts, mediates osteoclastogenesis and activates mature osteoclasts with RANK. As a feedback loop retro-control in this system, osteoblasts also secrete OPG, a RANKL decoy receptor, in order to inhibit osteoclast differentiation and activities and then bone resorption by blocking RANKL binding to RANK [8,12].

RANKL and RANK are expressed by several cancer cells and RANKL/RANK signaling has been shown to play a key role in cancer cell migration and tissue-specific metastatic behavior [11,13,14,15,16]. Moreover, aberrant RANKL expression induces uncontrolled bone destruction mediated by osteoclasts [17]. Osteosarcoma has been associated with a deregulated RANKL/OPG balance that leads to pathological bone features [18]. The RANKL/OPG ratio in the blood is increased in high-grade osteosarcoma, leading to the establishment of a vicious cycle between pathological bone remodeling and osteosarcoma growth [19]. RANK expression is not present in normal osteoblasts [20,21] and the veracity of RANK expression by osteoblastic tumor cells as osteosarcoma cells has long been debated. To date, most of the literature on this subject abounds in the sense of RANK expression, in patient biopsies as well as in most established osteosarcoma cell lines [20,22,23,24,25,26,27]. Consequently, RANKL can stimulate RANK-positive osteosarcoma cells, leading to specific biological processes such as cell migration that need to be deciphered. Interestingly, Chen et al., using a genetically-induced murine model for osteosarcoma, corresponding to targeted invalidations of *p53* and *Rb* in the osteoblast lineage [22], have reported that total invalidation of RANKL in these mice completely blocked tumor development, despite inducing osteopetrosis. This observation outlined the pivotal role played by active RANKL in tumor initiation [22].

The aim of the present study was to clarify the later role of the RANKL/RANK axis on tumorigenesis and metastasis processes using human and murine RANK-expressing osteosarcoma cell lines. RANK over-expressing cells were inoculated in various mouse strains (immune-competent, immune-deficient and RANKL invalided ubiquitously or specifically in T-cells) and the impacts on the main cell processes were scrutinized. A comparative analysis by tissue microarrays of RANKL, RANK and OPG expressions in the biopsies of patients with or without metastases at diagnosis was performed to link the preclinical data obtained to clinical evidence.

## 2. Results

### 2.1. Intrinsic RANK Expression by Osteosarcoma Cells Does Not Impact Cell Proliferation or Tumor Growth

**Figure 1:**
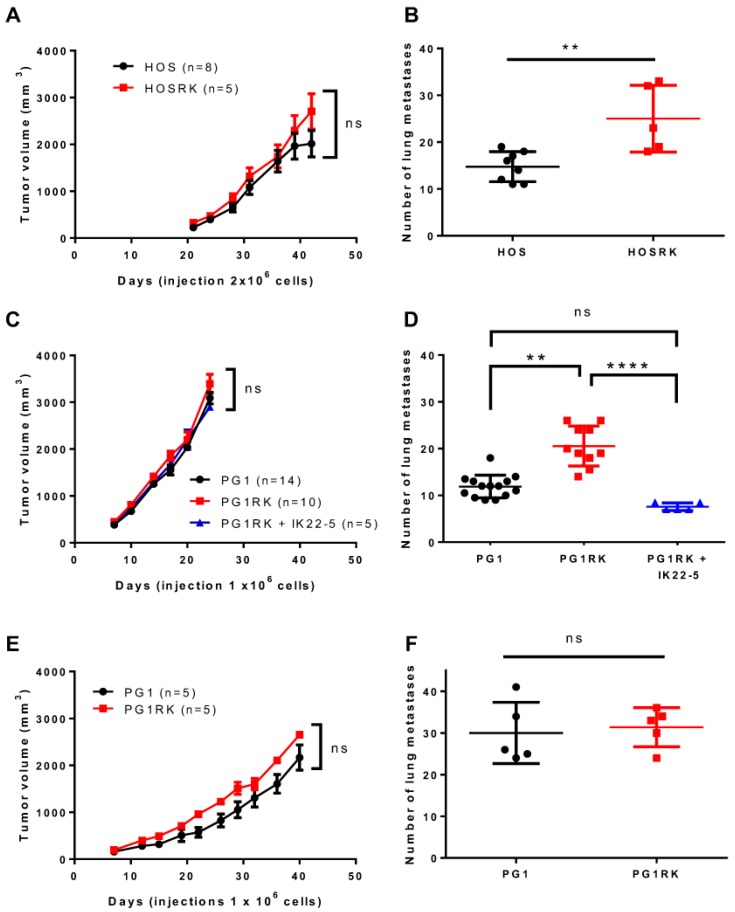
Impact of Receptor Activator of Nuclear factor κB (RANK) over-expression in osteosarcoma cells on tumor growth and the number of lung metastases. No significant difference was observed concerning tumor growth regardless of the cell-line considered (K-HOS (**A**), MOS-J PG1 (**C**,**E**)) or the immune status of the host mouse strain (Nude (**A**,**C**) or C57BL/6 (**E**)). However, regarding the number of lung metastases, a significant increase was observed regardless of the RANK over-expressing cell-line considered, in immune-deficient Nude mice (**B**,**D**) but not in immune-competent C57BL/6 mice (**F**). Moreover, injections of a Receptor Activator of Nuclear factor κB Ligand (RANKL)-blocking antibody (IK22.5) in Nude mice made it possible to reduce the number of lung metastases obtained with RANK expressing PG1 (**D**). n: number of mice in each group. Growth curves (**A**,**C**,**E**) are shown as the mean ± SEM. All data analysis was performed with the Kruskal Wallis test. ns: not significant; **: *p* < 0.01; ****: *p* < 0.0001.

**Figure 2:**
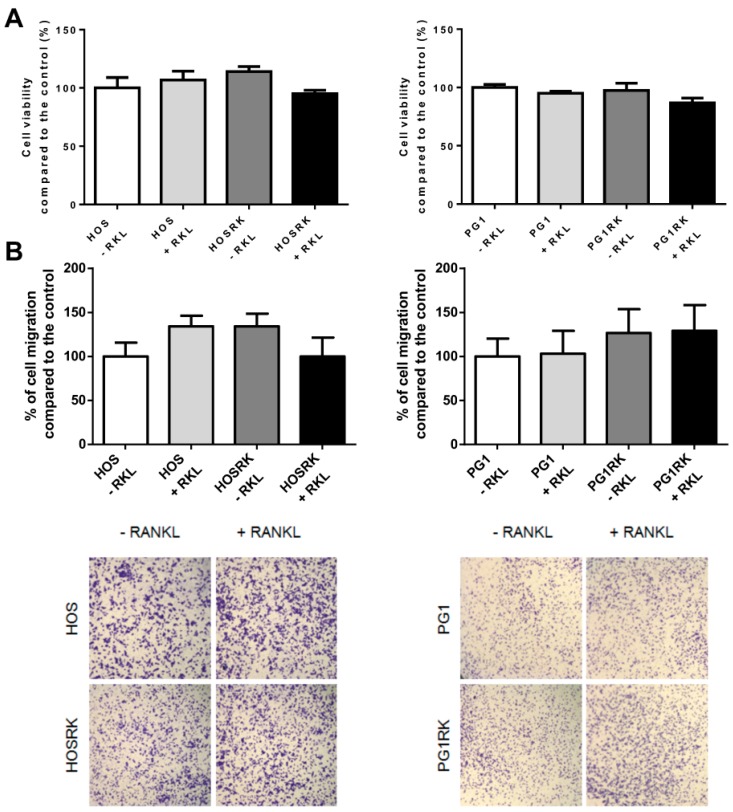
Consequences of RANK over-expression in osteosarcoma cells on cell viability (**A**) and migration (**B**). A moderate decrease (tendency) in the cell viability in response to the addition of RANKL to the culture medium was observed for both RANK over-expressing HOS cells and PG1 cells (**A**). For cell migration, which was evaluated with Boyden chambers, no significant impact of RANK over-expression and RANKL stimulation was observed, regardless of the cell-line considered (**B**).

RANK expression, in human KHOS (HOS) or mouse MOS-J PG1 (PG1) osteosarcoma cell lines, had no significant impact on tumor growth as assessed in NMRI Nude mice (Figure 1A,C). Similar observations were reported when PG1 cells were injected into C57BL/6 immune-competent mice (Figure 1E). However, significantly more rapid growth of PG1 tumors was observed, independently of RANK expression, in immune-compromised Nude mice compared to C57BL/6 mice (Figure 1C versus Figure 1E). These results were confirmed with MOS-J A3N cells (Figure S2). Immuno-histologic assessment of RANK and Ki67 expressions in tumors developed from injections of native and RANK over-expressing HOS cells, confirmed that RANK expression at the membrane surface had no incidence in vivo on the proliferation of tumor cells, as evidenced by Ki67 immunostaining (Figure S3). In order to strengthen these observations, cell viability was assessed in vitro with XTT assays. The results showed that RANK over-expression in HOS cells did not modify cell viability compared to the control cells (Figure 2A). However, while addition of soluble RANKL to native cells did not influence cell viability, RANKL seemed to induce a moderate (though not significant) decrease in the viability of RANK expressing HOS cell (Figure 2A). This slight tendency was also observed for MOS-J PG1 cells (Figure 2A).

Overall, these data confirmed that RANK expression by osteosarcoma cells had no major influence on cell proliferation or tumor growth.

### 2.2. RANK Expression by Osteosarcoma Cells Increases the Number of Lung Metastases Rankl-Dependently in Nude Mice

The migration capacity of RANK over-expressing HOS or MOS-J PG1 cells was assessed first in vitro (Figure 2B). RANK expression did not modulate the migration properties of osteosarcoma cells in either the presence or absence of RANKL. The migration ability of these RANK over-expressing cells was then analyzed in in vivo models. Injections of RANK over-expressing MOS-J PG1 cells in immune-competent mice did not impact the number of lung metastases compared to mice receiving non-modified cells (Figure 1F). These results were confirmed in a second set of experiments performed with MOS-J A3N cells (Figure S2D). On the contrary, the number of lung metastases increased significantly when RANK over-expressing cells were inoculated into immune-deficient mice (Figure 1D and Figure S2B). Interestingly, this increase was prevented by a treatment with the IK22-5 RANKL blocking antibody (Figure 1D and Figure S2B) while IK22-5 had no effect on the number of lung metastases occurring after injection of non-modified cells. Similar experiments performed with human HOS osteosarcoma cells confirmed that RANK over-expression led to a higher number of lung metastases in nude mice (Figure 1B).

Altogether, these experiments show that RANK over-expression by osteosarcoma cells RANKL-dependently favored the development of lung metastases in the Nude immune-compromised context.

### 2.3. RANK Expression by Osteosarcoma Cells Has No Impact on Tumor-Associated Bone Destruction

**Figure 3:**
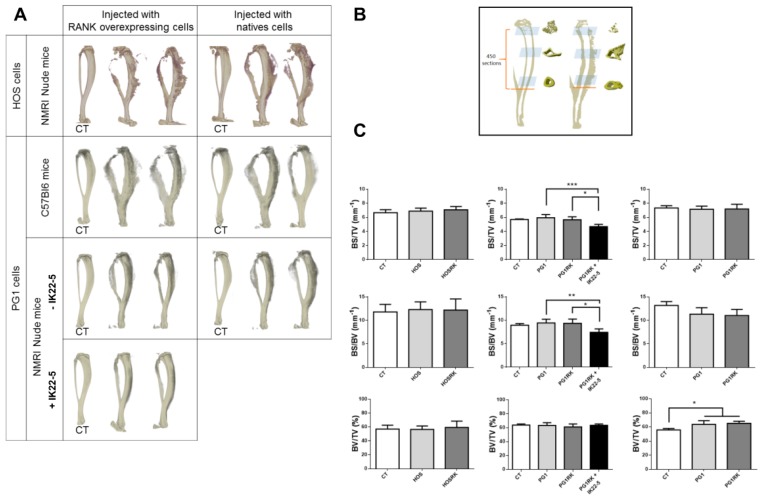
Impact of RANK over-expression in osteosarcoma cells on bone structure (**A**) and bone parameters (**B**,**C**). Whatever tumor cell-line or host mouse strain was considered, representative three dimension images did not make it possible to observe any difference concerning bone resorption and osteoid tissue formation in relationship with RANK over-expression (**A**). As expected, IK22-5 RANKL blocking antibody injections made it possible to protect the bone from resorption (**A**). The bone parameters BS/TV, BS/BV and BV/TV were measured on 450 sections whose positions are presented in (**B**). The results showed no difference between the cells over-expressing or not RANK, whatever parameter was considered (**C**). The IK22-5 RANKL blocking antibody made it possible to significantly reduce the BS/TV and BS/BV parameters, with no impact on the BV/TV parameter. A significant difference was observed concerning the BV/TV parameter only in C57BL/6 mice when comparing contra-lateral control tibias (CT) and tibias with tumors independently of RANK over-expression by tumor cells. The data in (**C**) are shown as the mean ± SD. Data analyses were performed using the Kruskal Wallis test. ns: no significant. *: *p* < 0.05. **: *p* < 0.01. ***: *p* < 0.001.

Injection of native osteosarcoma cells (HOS or MOS-J PG1) into the vicinity of mouse tibias caused dramatic bone destruction and tumor osteoid tissue formation (Figure 3A). RANK over-expression in these cells had no impact on these effects (Figure 3A,C). Interestingly, as expected from its major implication in osteoclastogenesis, RANKL blockage using the IK22-5 antibody significantly preserved bone from its tumor-induced destruction (Figure 3A,C) with the presence of a significant decrease in the BS/TV and BS/BV parameters (Figure 3C). Regardless of RANK status, in Nude mice the tumors had no effect on the BV/TV parameters, while a significant increase was observed in immune-competent C57BL/6 mice (Figure 3C). This increase may reflect greater osteoid tissue formation in immune-competent mice, which may be the consequence of slower tumor growth, thus providing a longer time for mineralization to occur.

### 2.4. Consequences of Adding RANKL to RANK Over-Expressing HOS Cells, with Regard to Expression Levels of the Genes Encoding Factors Implicated in the Different Cellular Processes

RANK over-expressing or native HOS cells were treated or not with sRANKL for 24 h and expression levels of the genes involved in proliferation, differentiation, migration, and oncogenic processes were assessed by RT-qPCR (Table 1). RANKL increased its own gene expression both in non-modified and RANK over-expressing cells, suggesting the existence of an autocrine stimulation loop. In contrast, no change in *OPG* and *RANK* expression levels was observed after the addition of RANKL. The functionality of RANKL/RANK signalization was confirmed by the significant increase in the expression level of two direct targets of this signalization, *NFATC1* and *NFκB*, only in RANK over-expressing cells stimulated with RANKL (Table 1). Regarding the expression of the markers involved in proliferation and apoptosis processes, RANKL up-modulated *p21* mRNA expression in RANK over-expressing cells, while no change in the transcript levels of *Bcl-2*, *Bax* and *p53* was observed (Table 1). In addition, transcript expression levels of undifferentiated cell markers or early differentiation markers such as *SOX9* and *COLL1a1* were down-regulated, whereas levels of late differentiation markers such as *OPN* and *BSP* were increased (Table 1). RANKL thus appeared to act as an autocrine pro-differentiation factor in RANK over-expressing cells.

In RANK over-expressing cells, the expression level of metalloproteinase genes such as *MMP9* and *MMP13* increased while *TIMP2* inhibitor gene expression slightly decreased (Table 1). Zymography experiments confirmed the impact on MMP9 activity, while MMP2 activity was unaffected (Figure S4). Altogether these results supported the pro-metastasis effect of adding RANKL to RANK over-expressing osteosarcoma cells.

The impacts of adding RANKL to certain oncogene expression levels were also analyzed on non-modified and RANK over-expressing HOS cells. A significant increase in *c-FLIP* and *c-MYC* gene expression was observed under RANKL stimulation, in contrast to non-modified cells (Table 1). *C-MET* expression in RANK over-expressing cells was unaffected by the addition of RANKL.

### 2.5. Genetically-Achieved RANKL Depletion in T Cells Has No Impact on Tumor Growth or Metastatic Spread

**Figure 4:**
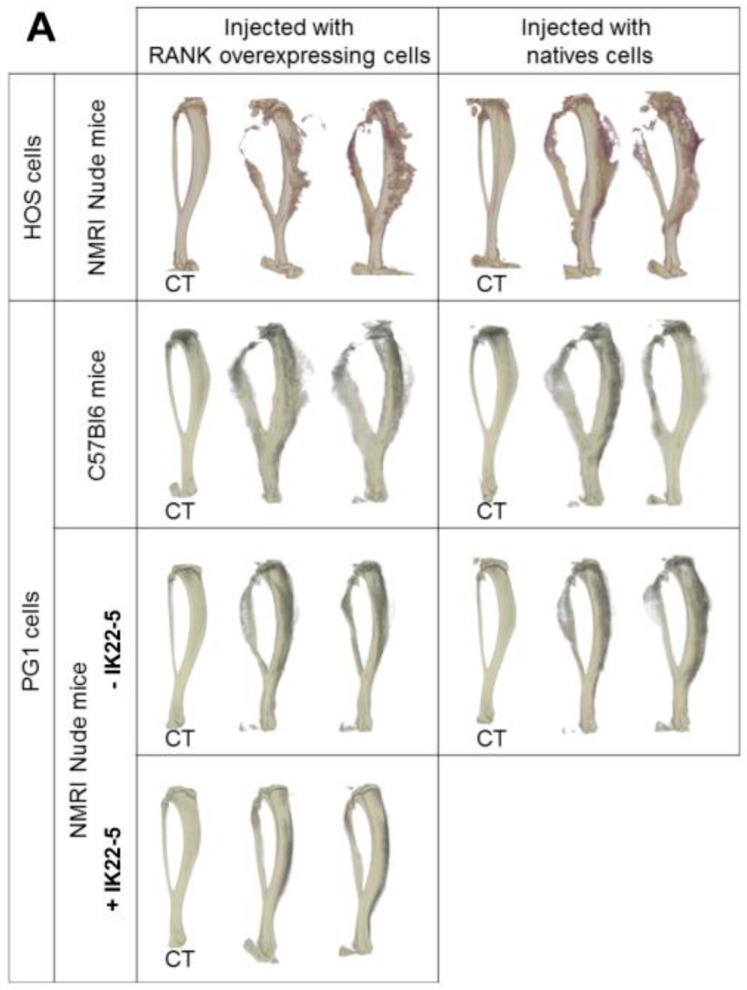

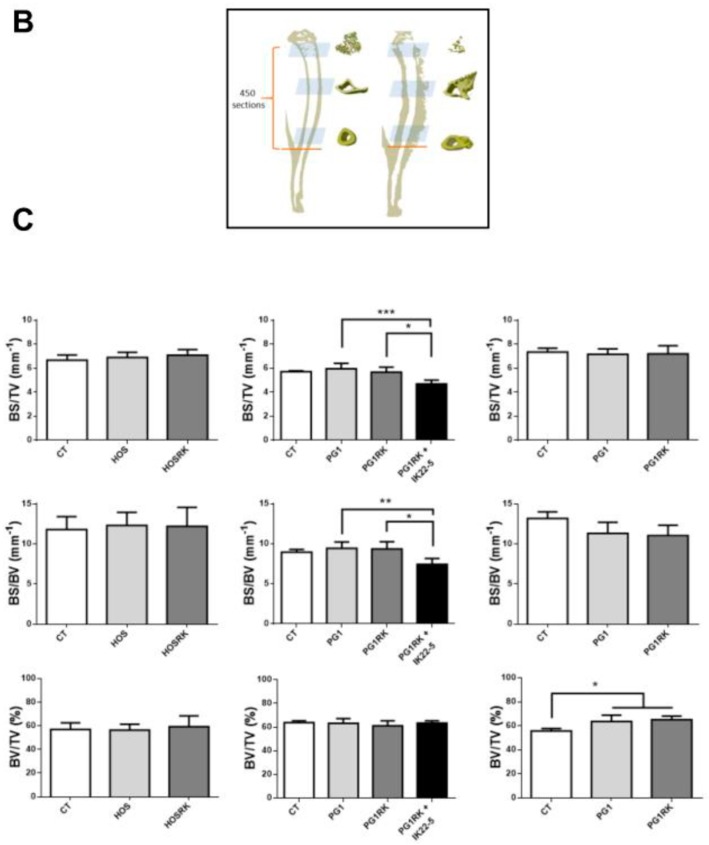
Consequences of T-cell specific invalidation of *Rankl* in recipient mice on RANK over-expressing PG1 tumor growth, number of metastases and bone parameters. *Rankl* depletion in mouse T-cells (LCK-CRE) was validated by PCR on gDNA extracted from tails and T cells (**A**). PCR data confirmed the effective recombination, specifically in T-cells. After injection of one million PG1 cells over-expressing RANK, tumor growth (**B**) and the number of lung metastases formed (**C**) were compared between mice invalidated for *Rank* in T-cells (*n* = 4) and control mice (*n* = 5) showing no difference. Representative three-dimensional images of tibias with tumors (and their controls, **C**) did not make it possible to observe any differences regarding bone resorption or tumor osteoid tissue formation (**D**). Micro-CT analysis of the BS/TV (mm-1), BS/BV (mm-1) and BV/TV (%) parameters of the tibias revealed no differences. However, an increase in BV/TV was observed comparatively to contralateral safe tibias, independently of the mouse genotype (**E**). The data in (**E**) are shown as the mean ± SD. Data analyses were performed using the Kruskal Wallis test. ns: no significant. *: *p* < 0.05.

In order to determine whether or not the increase in the number of metastases observed in Nude mice injected with RANK over-expressing osteosarcoma cells could be linked to the absence of RANKL expressing T-lymphocytes, RANKL*^Δ^*^T^ and control mice (Figure 4A) were injected with RANK over-expressing PG1 cells. Tumor growth (Figure 4B), the number of lung metastases (Figure 4C) and bone parameters (Figure 4D,E) appeared similar in both recombined and control mice.

### 2.6. Comparative Analysis of RANK, RANKL and OPG Expression in Biopsies of Patients with or without Metastases at the Time of Diagnosis

**Figure 5:**
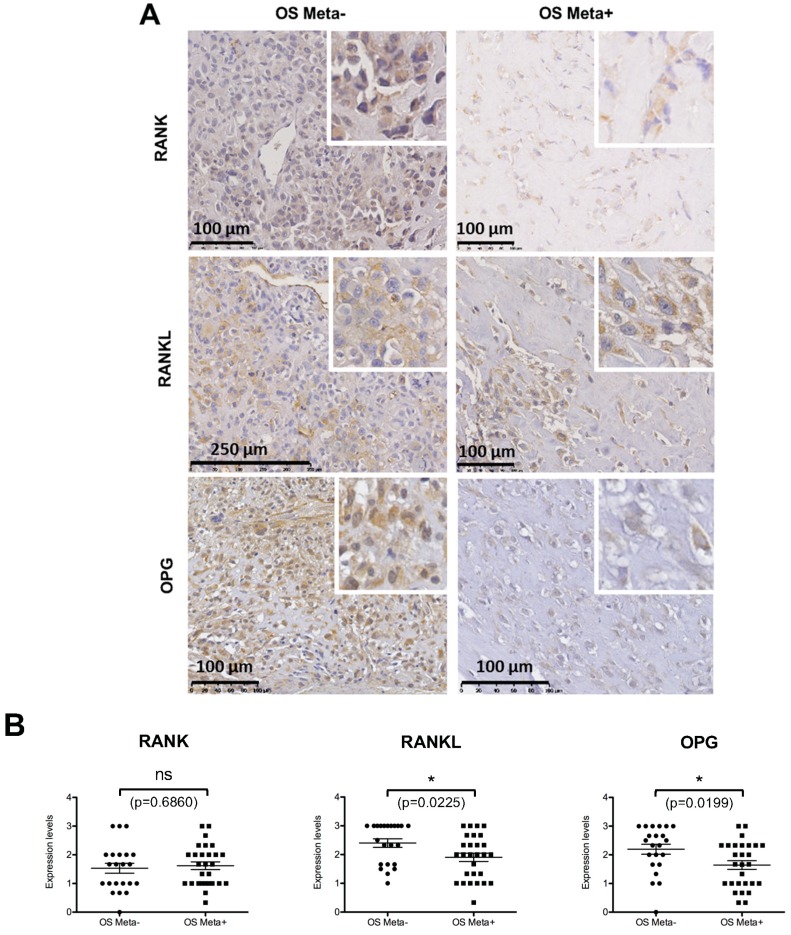

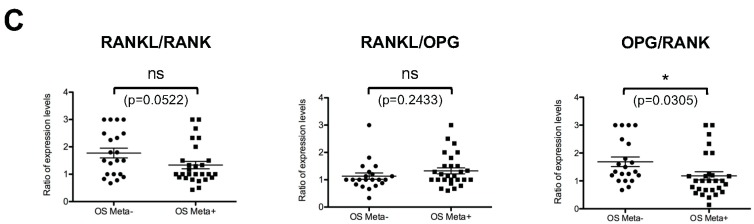
Tissue array analysis of RANK, RANKL and OPG expressions in a cohort of 50 biopsies of patients with (*n* = 28) and without (*n* = 22) metastases at time of diagnosis. Representative views of the different immuno-stainings are presented (**A**) with enlargement inset to clearly show the stained osteosarcoma cells. Statistical analyses (Student test) of the staining based on the percentage of stained osteosarcoma cells revealed a significantly lower number of positive cells for RANKL and OPG in the metastatic patient group, while no difference was observed between the two groups concerning the number of RANK-expressing cells (**B**). Statistical analysis of the three ratios between these factors, established individually for each patient, showed that only the OPG/RANK ratio was significantly different between the two groups with lower values in the metastatic patient group (**C**). ns: not significant. *: *p* < 0.05. *p* values are given for each test.

Tissue microarrays composed of the osteosarcoma biopsies of 50 patients (28 of them with metastases at the time of diagnosis) were performed for RANK, RANKL and OPG expression (Figure 5). RANK, RANKL and OPG expressions in osteosarcoma cells were observed in most of the biopsies analyzed (Figure 5A). No significant difference between patients with or without metastases was observed for RANK, while RANKL and OPG expressions were significantly lower in metastatic patients (Figure 5B). Interestingly, a significant decrease in OPG expression compared to RANK was observed in patients with metastases (Figure 5C), while ratios of RANKL with both receptors were not significantly different between the two groups of patients.

## 3. Discussion

**Figure 6:**
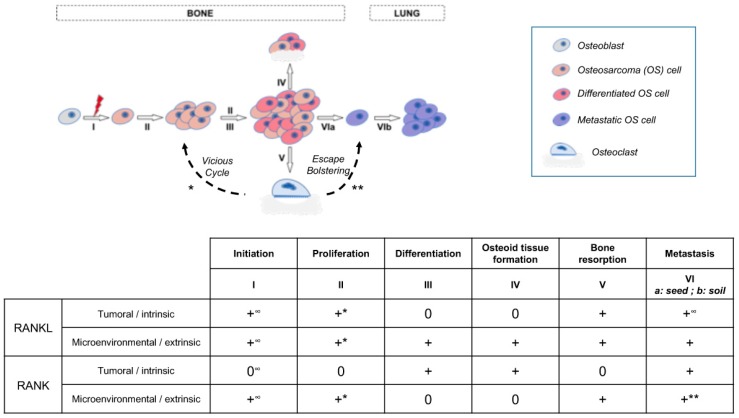
Schematic representation of the different stages of osteosarcoma from initiation to lung metastasis, and the implications of intrinsic and extrinsic RANKL and RANK in these different stages. +: implication revealed; 0: No implication; *: part of the vicious cycle; **: part of the escape bolstering; ∞: according to Chen et al., 2016.

The aim of the present work was to decipher the consequences of RANK expression in osteosarcoma cells on basic cell-processes such as proliferation, differentiation and migration. The data obtained showed that RANK expression by osteosarcoma cells had no direct effect on proliferation or tumor growth in various murine models despite observed increases in the expression of the anti-apoptotic oncogene c-Flip [28] and the pro-proliferation oncogene c-Myc [29]. The effect of the latter was potentially balanced by increased expression of P21. This absence of effect on proliferation was also reported with genetically-engineered murine models of osteosarcoma [22]. However, a positive effect of RANKL-RANK signaling on tumor growth has previously been reported for cancers in various organs including bone [17,23,30]. Consequently, our data suggest that this positive effect of RANKL/RANK signaling, at least for osteosarcoma, does not correspond to direct stimulation of tumor cell proliferation (intrinsic), but rather to an indirect effect with the implication of cells from the microenvironment (extrinsic) (Figure 6). A vicious cycle (Figure 6) was previously brought to light between tumor cells and RANKL-dependent bone resorbing osteoclasts [17,31]. Our data also showed that RANK expression by tumor cells has no consequence on bone resorption associated with tumor growth (Figure 3). This suggests an absence of connection between RANK expression in tumor cells and the vicious cycle implicating RANKL-dependent osteoclastic cells. To a greater extent, all the cells in the microenvironment that express elements of the RANKL/RANK/OPG triad may take part in the vicious cycle and modulate tumor growth [9]. Indeed, RANKL/OPG expressing cells in the bone microenvironment such as T- and B-lymphocytes, endothelial cells, stromal cells, and osteoblastic cells may take part in tumor growth. Further studies will thus be needed to decipher the implication of each RANKL expressing-cell from the bone microenvironment on osteosarcoma growth.

Despite the absence of direct effect of RANKL on the proliferation of RANK-expressing osteosarcoma cells, our data suggest the existence of a direct effect on the cell differentiation process. Adding RANKL to cultures of RANK-expressing osteosarcoma seems to boost osteosarcoma cell pseudo-differentiation to a more mature stage with respectively increased and decreased expressions of late and early osteoblast differentiation markers such as *BSP* and *RUNX2* (Table 1). This pro-differentiation function of RANKL on RANK-expressing cells has already been described for several healthy cells, including mammary glands and hair-follicle epithelial cells or pre-osteoclastic cells [32,33], but also in tumors derived from these cell-types, such as breast carcinoma and giant cell tumors in bone [34,35]. All these data underline that in either physiological or pathological contexts RANKL is capable of stimulating cellular differentiation (Figure 6) thanks to binding to RANK, regardless of the epithelial, mesenchymal or hematopoietic origins of the cell [9]. Interestingly, the RANKL-dependent pro-differentiation effect associated with RANK expression by osteosarcoma cells, which is normally synonymous in osteoblastic cells with an increase in osteoid tissue formation, was not correlated with any increase in bone parameters in our in vivo models. One explanation may be the compensation of this over-formation of osteoid tissue by the concomitant increase in bone resorption induced by RANKL in the tumor microenvironment regardless of the intrinsic or extrinsic origin (Figure 6). Interestingly, a relationship between osteosarcoma cell differentiation status and patient life-expectancy has been suggested [36]. In this context, RANK expression by osteosarcoma cells appears more as a factor of good prognosis according to the observations of a pro-differentiation effect of RANKL on these cells.

However, our data on the consequences of RANK expression on the metastatic process unbalanced this assertion. Indeed, in immune-compromised mice, RANK expression by tumor cells favors the occurrence of lung metastases. The metastatic dissemination promoting effect of RANKL/RANK signaling (Figure 6) had already been described for tumors with bone metastases such as breast, prostate and lung tumors [37,38,39] but the relationship with the immune-compromised environment is something new. Chemotherapies are known to induce immunosuppression [40], so in case of tumor cell resistance to treatment, if the cells express RANK, RANKL/RANK signaling may participate in the occurrence of metastases. As a result, regardless of the type of tumor, inhibition of this signaling may be of major relevance for preventing metastases. This assertion is supported by the fact that rare metastases are observed in RANKL null mutant mice injected with MOS-J PG1 cells (Figure S5), and by the observation that RANKL inhibition through a blocking antibody is capable of reversing the metastatic effect favored by RANK expression in osteosarcoma cells in an immune-compromised context. Interestingly, with our mouse model with para-tibiae injections, we have established that the source of RANKL modulating the metastatic process is not the T-lymphocytes as first hypothesized, based on observation of the RANKL-dependent increase in the number of lung metastases in Nude mice. The origin of RANKL in question remains an open question. The question raised is at what stage in the metastasis process RANKL is implicated. According to the stimulation of MMPs, RANKL may favor the escape of the tumor cell from its initial site, but involvement in seeding to the lung cannot be excluded. Interestingly, injecting PG1 cells into different microenvironments regarding the RANKL/RANK/OPG system (Figure S5) revealed that only invalidation of *Rankl* (*Rankl^-/-^*) had an impact on the number of lung metastases, independently of RANK expression by the osteosarcoma cells, while either *Opg* invalidation (*Opg^-/-^*) or *Rank* over-expression in the monocyte/macrophage lineage [*Rank^Tg^*, [33]] had no influence on this number compared to the WT microenvironment. These data suggest that the osteopetrotic microenvironment has an influence on the metastasis process of osteosarcoma independently of RANK expression by osteosarcoma cells, while an increase in the number of osteoclasts (osteoporotic microenvironment) has no bad influence on this process. Interestingly, this reinforces the value of using RANKL inhibition to treat osteosarcoma, but also sustains the role of osteoclasts in the metastasis process by bolstering tumor cell escape (Figure 6), as previously described [41]. Moreover, a decrease in PG1 cell-derived tumor growth was observed only in the *Rankl^-/-^* mice (Figure S5), reinforcing the importance of the vicious cycle in the growth of osteosarcoma (extrinsic, Figure 6), but also raising the question of other extrinsic implications of RANKL than osteoclastogenesis control. Interestingly, LGR4 was revealed as a third receptor for RANKL [42] and involvement of the RANKL-LGR4 axis cannot be excluded from tumor growth. LGR4 was shown to be expressed by osteosarcoma cells [43] and, in several forms of cancer, implicated in the tumor progression, migration and metastatic processes (for instance [44]. Consequently, in osteosarcoma cells, LGR4 may have a similar function in response to its ligand RANKL. Further studies will be needed to decipher the part of the RANKL-LGR4 axis in parallel to the RANKL-RANK axis in the ontogenesis of osteosarcoma.

In order to strengthen the data obtained in mice with regard to the involvement of intrinsic RANK signaling in the metastatic process of osteosarcoma, a retrospective study using immunohistochemistry was carried out on biopsies of a cohort of patients with or without metastases. The aim of this study was to reveal any correlations between relative expression levels of the different members of the RANK/RANKL/OPG triad and metastatic status. Firstly, the results showed that RANK was expressed in all the samples analyzed, at more or less variable rates, with no significant difference between patients with or without metastases. This data suggests that all patients have an equal risk of developing metastases if intrinsic RANK expression is considered a major pro-metastatic factor. This also suggests that the development of metastases is more determined by RANKL availability for binding to RANK. Interestingly, the OPG/RANK expression level ratio appears to be significantly decreased in patients diagnosed with metastases. In this context, RANKL available in the microenvironment may preferentially fix to the RANK expressed by osteosarcoma cells and stimulated by the metastatic process. RANK expression may thus be a predisposing factor for metastatic dissemination whose triggering element is a change in the RANK/OPG ratio.

## 4. Materials and Methods

### 4.1. Cell Culture

The murine osteosarcoma MOS-J cell line derived from a spontaneous mouse osteosarcoma was provided by Dr. Shultz [45]. The two subclones used in the experiments were derived from this cell line using the limited dilution technique. The first clone, MOS-J PG1 (named PG1), revealed a high proliferation rate, while the second, MOS-J A3N (named A3N), showed a low proliferation rate. These clones were grown in RPMI1640 medium (Lonza, Walkersville, MD, USA) supplemented with 5% fetal bovine serum (FBS; Hyclone, Logan, UT, USA) and a mix of 100 U/mL of penicillin and 100 µg/mL of streptomycin (Lonza). The human osteosarcoma KHOS/NP (R-970-5) cell-line (named HOS in the manuscript) was purchased at the American Type Culture Collection (ATCC, Manassas, VA, USA; CRL-1554 lot number 203927 (Reference F-14748)). HOS cells were cultured in DMEM (Lonza) supplemented with 10% FBS and a cocktail of antibiotics (Lonza).

### 4.2. Lentivirus Production and Osteosarcoma Cell Transduction

EX-O0007-Lv105 and EX-Mm24198-Lv105 OmicsLink™ Expression vectors (GeneCopoeia, Rockville, MD, USA) containing respectively human and mouse *Rank* cDNA were used to produce lentiviral particles. pEZ-Lv105-eGFP was used as the control. Lentiviruses were produced using packaging vectors as described by Dull et al. [46]. Details of the virus production and osteosarcoma cell transduction are given in the supplementary Materials and Methods.

### 4.3. Validation of Functional Rank Over-Expression in Osteosarcoma Cells

The functionality of RANK over-expressed in human (HOS) and murine (MOS-J sub-clones) osteosarcoma cells was validated using a variety of techniques, including western blot and flow cytometry (the data obtained for HOS are shown in Figure S1).

### 4.4. Cell Viability Assay

Two thousand cells per well were seeded into a 96-multiwell plate and cultured in appropriate medium containing 1% FBS, in the presence or absence of 100 ng/mL of soluble human RANKL or soluble murine RANKL (R&D System, Abingdon, UK). Cell viability was determined by the 3’-[1-(phenylaminocarbonyl)-3,4-tetrazolium]-bis(4-methoxy-6-nitro) benzene sulfonic acid hydrate (XTT) cell proliferation assay kit II (Roche Diagnostics, Mannheim, Germany) following the manufacturer’s recommendations. After 72 h of culture, XTT reagent was added to each well and incubated for 5 h at 37 °C. Absorbance was read at 490 nm using a 96-multiwell microplate reader (Wallac 1420 Victor 2, Perkin Elmer, Waltham, MA, USA). A manual trypan blue exclusion test was also performed at the same time.

### 4.5. RNA Isolation, Reverse Transcription and Quantitative PCR

Total RNA was extracted from the cell cultures carried out in 6-multiwell plates using Direct-Zol RNA miniprep (ZymoResearch, Irvine, CA, USA) according to the manufacturer’s instructions and stored at −80 °C until use. First-strand cDNA was synthesized starting from 3 µg of total RNA and using Maxima H Minus Reverse Transcriptase (Thermo Scientific, Waltham, MA, USA) with Random primers. Quantitative real time PCRs were performed on the equivalent of 20 ng of reverse-transcribed total RNA with 300 nM of each primer (Table S1) and SYBR Select Master Mix (Applied Biosystems, Foster City, CA, USA). The results were acquired and analyzed using the CFX96 real-time PCR detector system and its software (Bio-Rad, Marnes-la-Coquette, France). For each pair of primers, a standard curve and a negative control were realized in order to verify PCR efficiency. *Gapdh* and *B2M* were used as the internal controls to calculate relative amplification. Results were calculated using the comparative method of relative quantification.

### 4.6. Migration Assay

Cells were seeded at a density of 1 × 10^5^ cells/mL in the upper surface of a Boyden Chamber (size of the pores, 8 µm) (BD Biosciences, Franklin Lakes, NJ, USA) containing 0.7 mL of media (1% FBS +/− 100 ng/mL of soluble RANKL). The chamber was immersed in a 24-multiwell plate containing 0.7 mL of complete media (DMEM + 10% FBS for HOS cells or RPMI + 5% FBS for MOS-J cells). After 24 h of incubation at 37 °C, the cells on the upper filter that had not migrated through were removed by wiping with a cotton bud. The remaining cells were fixed in 1% (*v*/*v*) glutaraldehyde solution for 10 min at room temperature then washed twice with PBS (pH 7.2). Cells that had migrated through the filter were stained for 20 min using a 10% (*v*/*v*) purple crystal solution in water. After drying, the cells were visualized and counted in five microscopic fields using an AxioVision Camera (Zeiss, Marly-le-Roi, France). The number of migrated cells was measured using Image J software (National Institutes of Health, Bethesda, MD, USA) and was evaluated in three independent experiments (*n* = 6).

### 4.7. Mouse Models of Osteosarcoma

All procedures involving mice were conducted in accordance with the institutional guidelines of the French Ethical Committee (n° 1280.01 and 1281.01). Mice were housed under pathogen-free conditions at the Experimental Therapy Unit (Faculty of Medicine, Nantes, France). For injection and tumor monitoring, mice were anesthetized by inhalation of a combination of isoflurane/air (1.5%, 1 L/min). Tumor volume (V), measured twice a week, was calculated using the formula: length × width × depth × 0.5. Data points were expressed as average tumor-volume ± S.E.M. Mice were sacrificed as soon as the tumor volume reached 2500–3000 mm^3^ (10% of body weight) for ethical reasons. The number of macroscopic lung metastases was counted manually for each mouse. The MOS-J models were induced in six different mouse strains (C57BL6/J immune-competent, NMRI-nude immune-deficient, *Rankl^Flox/Flox^ Lck-Cre* (*Rankl^ΔT^*), *Rankl^-/-^*, *Opg^-/-^* and *Rank^Tg^*) by an intramuscular injection of 1 × 10^6^ PG1 cells or RANK over-expressing PG1 cells in a paratibial site at the age of 5 weeks. The HOS model was induced similarly by inoculating 2 × 10^6^ HOS cells or RANK over-expressing HOS cells into 5-week-old female NMRI-Nude mice (Elevages Janvier, Le Genest Saint Isle, France). *Rankl^ΔT^* mice were obtained by crossing mice with a *Rankl* conditional allele (*Rankl^F/F^*) [10] and B6.Cg-Tg(*Lck-cre*)548Jxm/J mice (The Jackson Laboratory, Bar Harbor, ME, USA) to delete *Rankl* in the T-lymphocytes. *Rankl^-/-^* and *Rank^Tg^* mice were obtained as previously described [21,47]. *Opg^-/-^* mice were obtained from The Jackson Laboratory (Bar Harbor, ME, USA). Genotyping of the different transgenic mice is presented in the Supplementary Materials and Methods.

### 4.8. IK22-5 RANKL Blocking Antibody Injections

The mice were injected every three days subcutaneously with 75 µg of the IK22.5 RANKL blocking antibody [48], starting 5 days after inoculation of the tumor cells. The control animals were injected with physiologic serum.

### 4.9. Micro-CT Analysis

Analyses of bone microarchitecture were performed using a Skyscan 1076 in vivo micro-CT scanner (Skyscan, Kontich, Belgium). Tests were performed after sacrifice on tibias for each group. All tibias were scanned using the same parameters (pixel size 18 µm, 50 kV, 0.5-mm Al filter, 10 min of scanning). The reconstruction was analyzed using NRecon and CTan software (Skyscan). The following bone parameters were measured: tissue volume (TV, mm^3^), bone volume (BV, mm^3^), percentage of bone volume (BV/TV, %), tissue surface (TS, mm^2^), bone surface (BS, mm^2^), bone surface/bone volume ratio (BS/BV, mm^3^), bone surface density (BS/TV, mm^3^), trabecular spaces (Tb.Sp, mm) and cortical or trabecular thickness (CTh, Tb.Th, mm).

### 4.10. Immunohistochemistry

After euthanasia, tumor samples were preserved and fixed in 4% of PFA, decalcified with 4.13% of EDTA, and 0.2% of PFA in PBS using a microwave tissue processor (KOS, Milestone, Kalamazoo, MI, USA) for 4 days and embedded in paraffin. Three micrometer-thin sections were then stained for human KI67 (1/100, M7240, Dako, Agilent, Santa Clara, CA, USA) and RANK (1/50, Sc-9072, Santa-Cruz, Heidelberg, Germany).

### 4.11. Tissue Microarrays

Tissue microarrays (TMAs) were prepared from biopsies of a cohort of 50 patients with osteosarcoma from Nantes University Hospital as previously described [49]. At the time of diagnosis, the presence of metastases was observed in 28 of these patients. Briefly, TMAs were prepared with three core samples measuring 1 mm in diameter for each case in the most representative areas included in paraffin blocks and cut into 3 µm sections. Immunohistochemistry was performed with antibodies against RANK (Goat polyclonal antibody AF683 from R&D Systems (Abingdon, UK), dilution 1/20), RANKL (Goat polyclonal antibody AF626 from R&D Systems dilution 1/20) and OPG (Rabbit polyclonal antibody Ab9986 from Abcam, Cambridge, UK, dilution 1/50) following the protocol previously described [49]. The TMA slides were scanned and the images were automatically digitized (Nanozoomer, Hamamatsu Photonics, Shizuoka, Japan) before quantification. Immuno-reactivity was analyzed qualitatively (cell type, location, nuclear/membrane/cytoplasmic staining) and semi-quantitatively. Semi-quantification was done for RANK, RANKL and OPG positivity using the following criteria: 0, no osteosarcoma cells stained; 1, <1/3 of the osteosarcoma cells stained; 2, between 1/3 and 2/3 of the osteosarcoma cells stained; 3, >2/3 of the osteosarcoma cells stained. For each case, the mean of the values in the three core samples was calculated for further statistical analyses. For observations and semi-quantifications, a double-blind examination by two experienced pathologists was carried out.

### 4.12. Statistics

The differences between the experimental conditions were assessed with Student’s *t* test or a one-way ANOVA followed by the Mann–Whitney test. The results are given as a mean ± SEM or SD from at least three independent experiments. Results were considered significant at *p* values < 0.05. GraphPad Prism 6 (GraphPad Software, San Diego, CA, USA) software was used for all statistical analyses.

### 4.13. Western Blot Analysis, Zymography Assay and Flow Cytometry 

These are presented in the Supplementary Materials and Methods as Corresponding to the Supplementary Figures.

## 5. Conclusions

RANKL has previously been implicated in the initiation of osteosarcoma. The present work was devoted to analyzing other potential implications of the RANKL-RANK axis in osteosarcoma based on the expression of RANK by the tumor cells (intrinsic expression). The data obtained showed that RANKL was also able to stimulate the pseudo-differentiation of osteosarcoma cells and to stimulate the metastatic process in an immune-compromised context. In addition, RANKL can stimulate tumor growth but through an indirect effect that involved at least osteoclasts in a process known as the “vicious cycle”. Altogether, the present data on the involvement of the RANKL-RANK axis in osteosarcoma plead in favor of targeting this axis for the treatment of these highly metastatic tumors.

## Figures and Tables

**Table 1:** RT-qPCR analysis of the impact of adding RANKL to cultures of HOS cells that are native or over-expressing RANK.

Genes	Fold Change HOS + RKL vs. HOS	Fold Change HOSRK + RKL vs. HOSRK
*RANK*	1.10 ± 0.18	1.58 ± 0.14
*RANKL*	4.44 ± 1.99	77.94 ± 28.37
*OPG*	1.00 ± 0.10	1.09 ± 0.12
Apoptosis and proliferation		
*Bax*	1.18 ± 0.12	1.21 ± 0.11
*Bcl2*	−1.04 ± 0.23	1.28 ± 0.15
*p21*	1.18 ± 0.21	4.22 ± 0.57
*p53*	1.05 ± 0.08	−1.23 ± 0.12
Differentiation		
*RUNX2*	1.01 ± 0.07	−1.28 ± 0.54
*SOX9*	−1.17 ± 0.07	−2.14 ± 0.05
*COLL1a1*	−1.09 ± 0.07	−6.00 ± 0.05
*OCN*	1.70 ± 0.14	1.41 ± 0.15
*OPN*	1.58 ± 0.12	2.81 ± 0.42
*BSP*	−1.06 ± 0.32	50.25 ± 20.44
Migration		
*MMP2*	−1.31 ± 0.16	−1.43 ± 0.06
*MMP9*	−1.03 ± 0.14	7.98 ± 0.97
*MMP13*	1.24 ± 0.30	11.73 ± 1.41
*TIMP1*	1.07 ± 0.08	1.07 ± 0.12
*TIMP2*	−1.05 ± 0.13	−1.38 ± 0.11
Oncogenes		
*c-FLIP*	−1.23 ± 0.07	2.59 ± 0.44
*c-MET*	−1.13 ± 0.10	−1.11 ± 0.25
*c-MYC*	−1.17 ± 0.07	3.58 ± 0.31
Others		
*NFATC1*	1.20 ± 0.14	7.13 ± 3.48
*NFkB*	1.05 ± 0.22	2.07 ± 0.53
*BMP2*	1.21 ± 0.35	3.24 ± 0.51
*TGF*b*1*	1.01 ± 0.07	1.13 ± 0.20

The cells were treated for 24 h with RANKL (100 ng/mL). Fold change represents the values related to untreated controls obtained after normalization by *B2M* and *Gapdh*. The data are presented as a mean ± SD (*n* = 6). Gray boxes correspond to genes revealing a fold-change induction, or reduction (−) of a factor of more than 2.

## Supplementary Materials

The following are available online at http://www.mdpi.com/2072-6694/10/11/398/s1, Figure S1: Validation of RANK forced-expression functionality in HOS cells, Figure S2: Injections of MOS-J A3N clone cells over-expressing RANK or not in C57BL/6 immune-competent and NMRI-nu immune-deficient mice, Figure S3: Analysis of the expression of RANK and Ki67 proteins using immunohistochemistry in sections of tumors induced by injections in NMRI-nu immune-deficient mice of HOS osteosarcoma cells that were native or over-expressing RANK, Figure S4: Analysis by zymography of the impact of adding RANKL to the culture medium of native or RANK over-expressing HOS cells on the activity of metalloprotease 2 and 9, Figure S5: Impact of the host mice’s genetic background on MOS-J PG1 tumor growth, the number of lung metastatic nodules and tumor osteoid tissue formation, Table S1: Sequences of primers used for RT-qPCR analysis. Supplementary Materials and Methods of Section 4.13.
