# Supplementary material for: The Intrinsic and Extrinsic Implications of RANKL/RANK Signaling in Osteosarcoma: From Tumor Initiation to Lung Metastases
Source: Cancers (Basel). 2018 Oct 24;10(11):398. doi: 10.3390/cancers10110398 (PMC6265790; doi:10.3390/cancers10110398)
Supplement: Supplementary file 1 [file cancers-10-00398-s001.pdf]

Supplementary Materials: The Intrinsic and Extrinsic Implications of RANKL/RANK Signaling in Osteosarcoma: From Tumor Initiation to Lung Metastases

Benjamin Navet, Kosei Ando, Jorge William Vargas-Franco, Régis Brion, Jérome Amiaud, Kanji Mori, Hideo Yagita, Christopher G. Mueller, Franck Verrecchia, Clotilde Dumars, Marie-Françoise Heymann, Dominique Heymann and Frédéric Lézot

1. Supplementary Figures

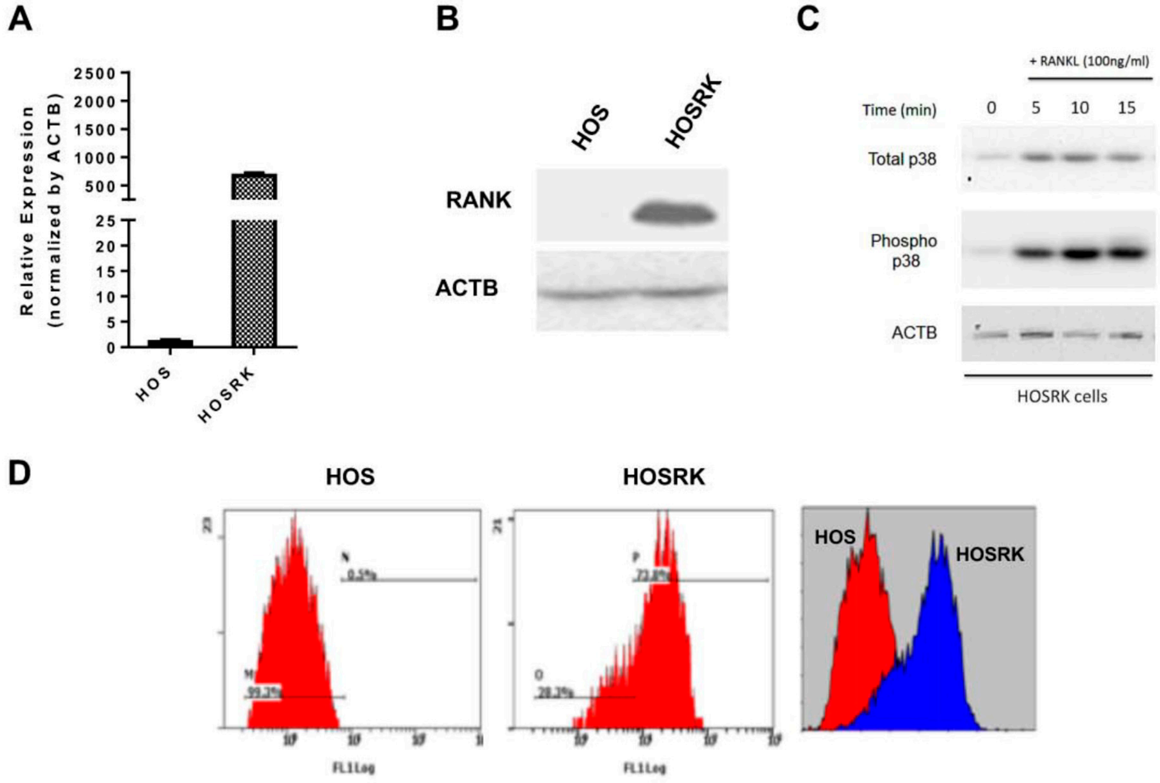

Figure S1. Validation of RANK forced-expression functionality in HOS cells by (**A**) qPCR analysis of RANK transcript expression, (**B**) Western blotting for RANK on 40 µg of total protein extract, (**C**) Western blotting for phosphorylated and total P38 on 40 µg of total protein extracted at different time-courses after addition of RANKL at 100 ng/mL and (**D**) flow cytometry analysis of RANK with AF682 antibody. The data showed that the *Rank* expression construction was well expressed (**A**) and induced significant expression of the encoded receptor at the cell surface (**B** and **D**). Moreover, this receptor was functional as attested by the rapid P38 activation following addition of RANKL to the cell culture medium (**C**).

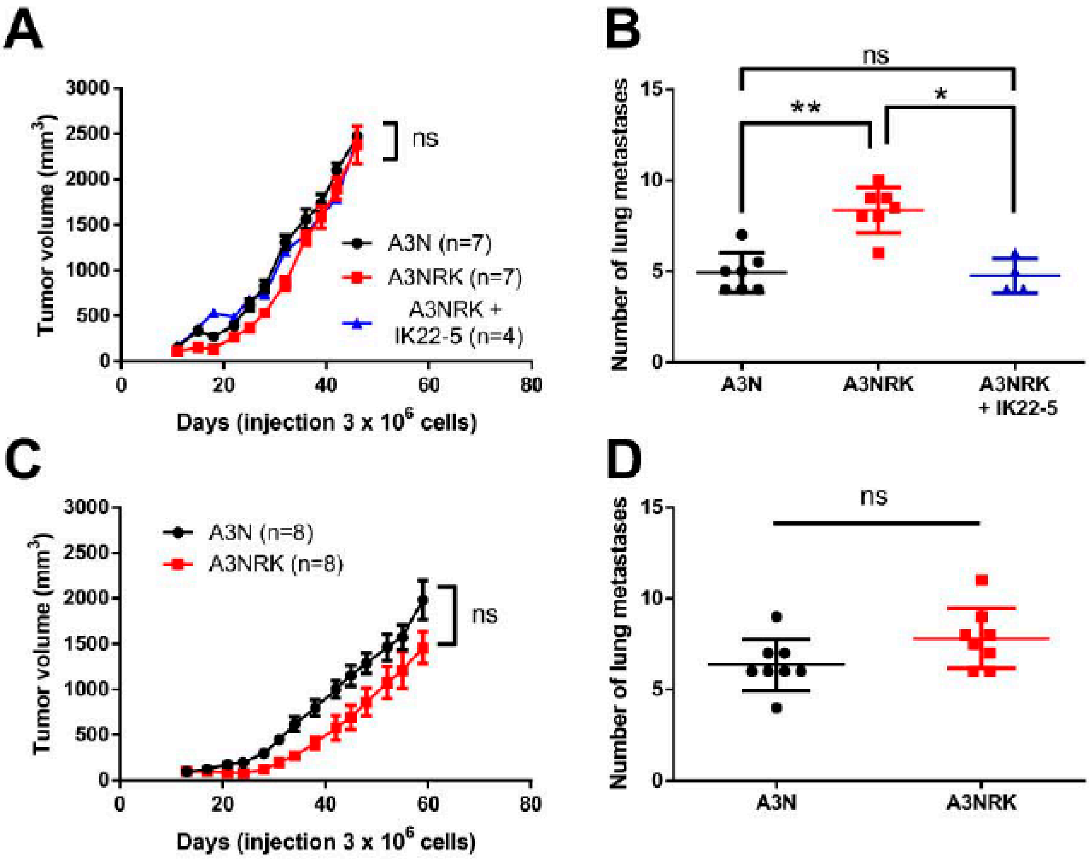

Figure S2. Injections of MOS-J A3N clone cells over-expressing RANK or not in C57BL/6 immune-competent and NMRI-nu immune-deficient mice. Tumor growth (**A**) and number of lung metastases (**B**) in NMRI nude mice after injection of three million A3N cells over-expressing RANK or not (7 mice in each group). A supplementary group ($n = 4$) injected with A3N cells over-expressing RANK was treated with an antibody blocking RANKL (IK22.5). The expression of RANK in A3N cells had no consequence on the tumor growth in the NMRI nude mice (**A**) but significantly increased the number of lung metastases (**B**). Moreover, this augmentation was reversed/prevented by the use of a RANKL blocking antibody (**B**). Tumor growth (**C**) and number of lung metastases (**D**) in C57BL/6 mice after injection of three million A3N cells over-expressing RANK or not (8 mice in each group). The expression of RANK in A3N cells had no consequence on either the tumor growth or the number of lung metastases in C57BL/6 mice. Growth curves (**A** and **C**) are shown as the mean ± SEM. All data analysis was performed using the Kruskal Wallis test: ns, not significant; * $p < 0.05$; ** $p < 0.01$.

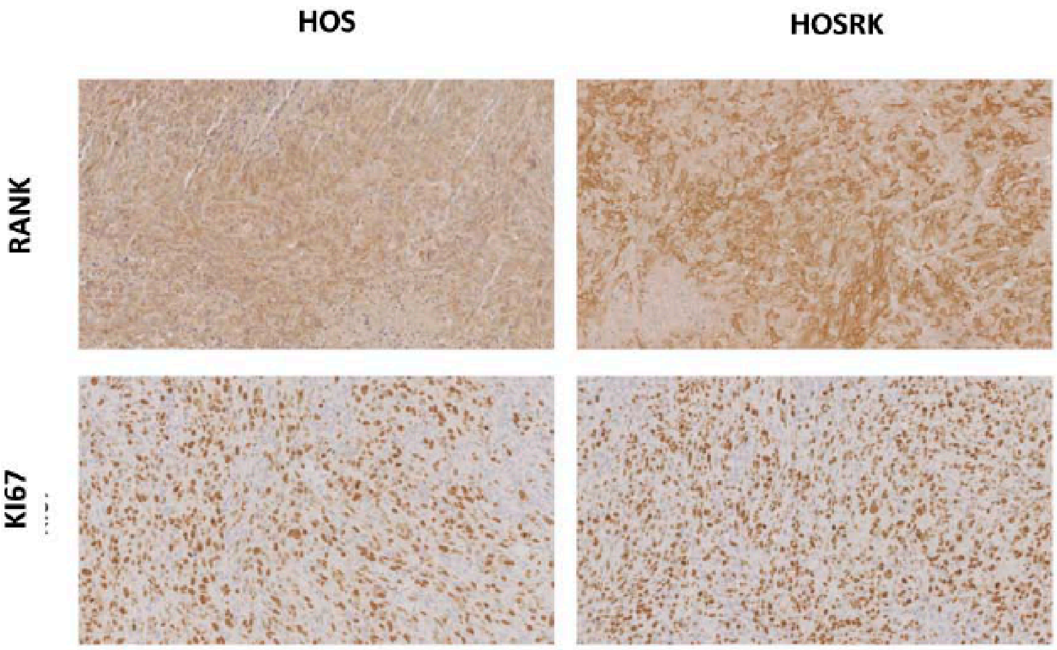

Figure S3. Analysis of the expression of RANK and Ki67 proteins using immunohistochemistry in sections of tumors induced by injections in NMRI-nu immune-deficient mice of HOS osteosarcoma cells that were native or over-expressing RANK. The RANK immunostaining appeared, as expected, more significant in the RANK overexpressing cells than in the parental cells while no difference in either the number of stained cells or the intensity of the staining was observed concerning the Ki67 immunostaining. Magnification ×200.

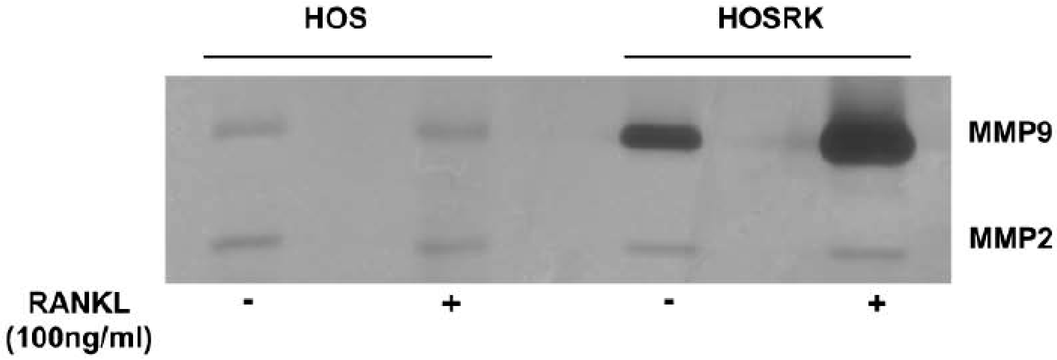

Figure S4. Analysis by zymography of the impact of adding RANKL to the culture medium of native or RANK over-expressing HOS cells on the activity of metalloprotease 2 and 9. In the absence of RANKL addition, RANK over-expression in HOS cells induced an increase in MMP9 activity, while MMP2 activity was unchanged. The addition of RANKL had no impact on the activity of either enzymes in native HOS cells, where as in the RANK overexpressing HOS cells significant stimulation of the MMP9 activity was observed. MMP2activity did not appear to be modulated by adding RANKL to these cells.

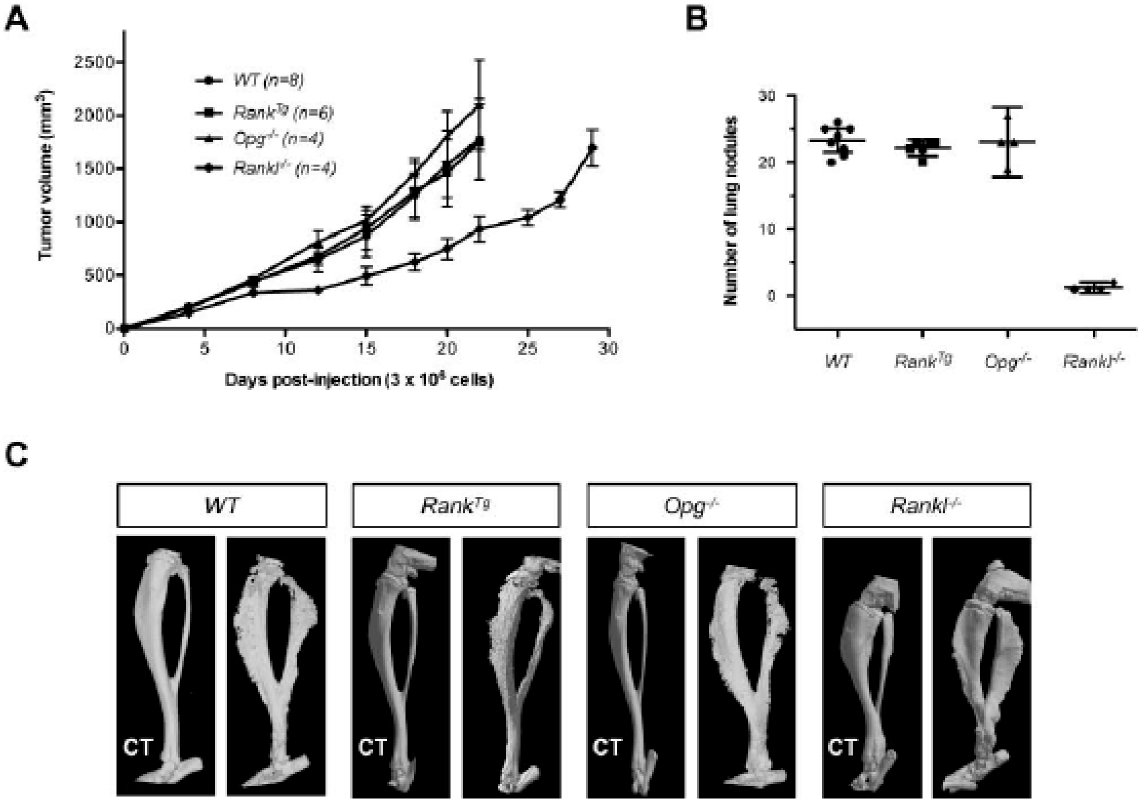

Figure S5. Impact of the host mice's genetic background on MOS-J PG1 tumor growth, the number of lung metastatic nodules and tumor osteoid tissue formation. MOS-JPG1 cells (3.106 cells) were injected into the vicinity of the tibias of 5-week-old *Ranktg*, *Opg*$^{-/-}$, *Rankl*$^{-/-}$ and wild type mice in order to analyze the impact of different genetic backgrounds regarding elements in the RANKL/RANK/OPG triad onto the tumor growth (**A**), the number of lung nodules (**B**) and the tumor osteoid tissue formation (**C**). Tumor growth appeared to be slowed down only in the *Rankl*$^{-/-}$ mice compared with the WT mice (**A**). Concerning the number of lung nodules, despite the longer time spent reaching the maximum ethically acceptable tumor volume (mouse sacrifice), *Rank*$^{-/-}$ mice revealed very few lung nodules compared to all other mice (**B**). For tumor osteoid tissue formation, significant apposition was observed in all genetic backgrounds in comparison with contralateral tibias (CT). However, this formation appeared less visible in osteolytic (*Ranktg* and *Opg*$^{-/-}$) backgrounds compared to WT, and more visible in the osteopetrotic (*Rankl*$^{-/-}$) background in relation to the different levels of osteoclast activity (**C**). n: number of mice.

2. Supplementary Tables

Table S1. Sequences of primers used for RT-qPCR analysis.

Gapdh	Forward	5′-TGGGTGTGAACCATGAGAAGTATG-3′
	Reverse	5′-GGTGCAGGAGGCATTGCT-3′
B2M	Forward	5′-TTCTGGCCTGGAGGCTATC-3′
	Reverse	5′-TCAGGAAATTTGACTTTCCATTC-3′
Rank	Forward	5′-TTCTGCTTCTCTTCGCGTCT-3′
	Reverse	5′-CCAGTGCCACAAATTAGCTGT-3′
Rankl	Forward	5′-TCGTTGGATCACAGCACATCA-3′
	Reverse	5′-TATGGGAACCAGATGGGATGTC-3′
Opg	Forward	5′-CAGCTCACAAGAACAGACTTTCC-3′
	Reverse	5′-TCGAAGGTGAGGTTAGCATGTC-3′
Bax	Forward	5′-ATGCGTCCACCAAGAAGC-3′
	Reverse	5′-ACGGCGGCAATCATCCTC-3′
Bcl2	Forward	5′-CTGCACCTGACGCCCTTCACC-3′
	Reverse	5′-CACATGACCCCACCGAACTCAAAGA-3′
p21	Forward	5′-CGAAGTCAGTTCCTTGTGGAG-3′
	Reverse	5′-CATGGGTTCTGACGGACAT-3′

p53	Forward	5′-ACATGACGGAGGTTGTGAGG-3′
	Reverse	5′-CGCAAATTTCCTTCCACTCGG-3′
Runx2	Forward	5′-GCCTAGGCGCATTTCAGA-3′
	Reverse	5′-GCTCTTCTTACTGAGAGTGGAAGG-3′
Sox9	Forward	5′-GTACCCGCACTTGCACAAC-3′
	Reverse	5′-TCGCTCTCGTTCAGAAGTCTC-3′
Coll1A1	Forward	5′-GATCCAGACTCTGACCTTTTGC-3′
	Reverse	5′-GCTCCAGCCTCTCCATCTTT-3′
Ocn	Forward	5′-GGCGCTACCTGTATCAATGG-3′
	Reverse	5′-GTGGTCAGCCAACTCGTCA-3′
Opn	Forward	5′-GCCGAGGTGATAGTGTGGTT-3′
	Reverse	5′-TGAGGTGATGTCCTCGTCTG-3′
Bsp	Forward	5′-CAATCTGTGCCACTCACTGC-3′
	Reverse	5′-CAGTCTTCATTTTGGTGATTGC-3′
Mmp2	Forward	5′-ATAACCTGGATGCCGTCGT-3′
	Reverse	5′-TCACGCTCTTCAGACTTTGG-3′
Mmp9	Forward	5′-GAACCAATCTCACCGACAGG-3′
	Reverse	5′-GCCCCAGAGATTTCGACTC-3′
Mmp13	Forward	5′-CCTGGACAAGTAGTTCCAAAGG-3′
	Reverse	5′-GCCGGTGTAGGTGTAGATAGGA-3′
Timp1	Forward	5′-GGGCTTCACCAAGACCTACA-3′
	Reverse	5′-TGCAGGGGATGGATAAACAG-3′
Timp2	Forward	5′-AGAAGAGCCTGAACCACAGG-3′
	Reverse	5′-TGACCCAGTCCATCCAGAG-3′
c-Flip	Forward	5′-CAGGAACCCTCACCTTGTTT-3′
	Reverse	5′-CAGATTTATCCAAATCCTCACCA-3′
c-Met	Forward	5′-TCTGCCTGCAATCTACAAGG-3′
	Reverse	5′-ATTATTCCTCCGAAATCCAAAGT-3′
c-Myc	Forward	5′-CACCAGCAGCGACTCTGA-3′
	Reverse	5′-GATCCAGACTCTGACCTTTTGC-3′
Nfatc1	Forward	5′-ATGAAGTCAGCGGAGGAAGA-3′
	Reverse	5′-GTGTGGAGGTCTGAAGGTTGT-3′
NfKb	Forward	5′-ATAATGCCTTCCGGCTGAGT-3′
	Reverse	5′-CCTCCACCAGCTCTCTGACT-3′
TGFB1	Forward	5′-GAGCCCAAGGGCTACCAT-3′
	Reverse	5′-GGGTTATGCTGGTTGTACAGG-3′
Bmp2	Forward	5′-AGGACCTGGGGAGCAGCAA-3′
	Reverse	5′-GCTCTTTCAATGGACGTGTCCC-3′

3. Supplementary Materials and Methods

3.1. Lentivirus Production and Osteosarcoma Cell Transduction

Briefly, 6 × 10^6 HEK293FT cells (human embryonic kidney cells optimized for viral production) were seeded and transfected 24 h later with 3 µg of each packaging plasmid (ViraPower™Packaging Mix, K497500; Invitrogen) and 9 µg of the transgene of interest (pEZ-Lv105-eGFP, EX-O0007-Lv105 and EX-Mm24198-Lv105); virus-containing supernatants were collected 48 h post-transfection and concentrated 60-fold by ultrafiltration. For titration, serial dilutions of the supernatants containing virus were tested on HEK293FT cells that were analyzed for EGFP expression 4 days post-infection using flow cytometry (FC500 flow cytometer, Beckman Coulter, Villepinte, France). The titers obtained were between 10^7 and 10^8 viral particles/µL. To generate stably modified HOS and MOS-J cell lines, 2 × 10^4 osteosarcoma cells were seeded in a 24-wells plate containing 300 µL of medium and infected with a multiplicity of infections of 50 particles/cell. After transduction, cells expressing the transgene were selected at confluence with 5 µg/mL puromycin (Gibco, Grand Island, NY, USA) to obtain a stable population. The eGFP and RANK expression levels were controlled using flow cytometry and RT-qPCR (for RANK).

3.2. Transgenic Mouse Genotyping

PCR was used to genotype the *Rankl*$^{-/-}$ mice with the following primers *5′-Rankl*: CCA AGT AGT GGA TTC TAA ATC CTG, *3′-Rankl*: CCA ACC TGT GGA CTT ACG ATT AAA G and *3′-insert*: ATT CGC AGC GCA TCG CCT TCT ATC. PCR was used to genotype the *Opg*$^{-/-}$ mice with the following primers *5′-Opg*: TGC CCT GAC CAC TCT TAT ACG GAC, *3′-Opg*: GGT CCT CCT TGA TTT TTC TAT GCC and *5′-insert*: TGA CCG CTT CCT CGT GCT TTA C. PCR was used to genotype the *Rank*^{Tg} mice with the following primers *5′-Rank*^{Tg}: ATG GAC TAC AAA GAC GAT GAC GAC A and *3′-Rank*^{Tg}: TGC CAG GAA TCC ACC GCC ACC AG. *Rankl*$^{Flox/Flox}$ *Lck-Cre* mice were genotyped for the *Rankl-flox* allele using the *5′-Rankl-Flox:* CTG GGA GCG CAG GTT AAA TA and *3′-Rankl-Flo*x: GCC AAT AAT TAA AAT ACT GCA GGA AA primers and for the *iCre* using the *5′-iCre*: ATG GTG CCC AAG AAG AAG AG and *3′-iCre*: CAG GTG CTG TTG GAT GGT CT primers.

PCR was performed on 1µL of gDNA from tails obtained with a Phire Animal Tissue Direct PCR Kit (Thermo Scientific, Waltham, MA, USA) according to the manufacturer's instructions. Recombination of *Rankl* in T cells was checked after gDNA purification of splenic T-lymphocytes. Briefly, spleen was collected and maintained in cold isolation buffer (PBS with 2% FBS, 2mM EDTA) before being treated with a Dynabeads® FlowComp™ (Thermo Scientific) Mouse Pan T (CD90.2) kit according to the manufacturer's instructions. Before performing genomic DNA extraction using a PureLink® Genomic DNA Mini Kit, sample purity was verify using flow cytometry with anti CD3 immunostaining (Clone 17A2, ref555274, BD Biosciences, Franklin Lakes, NJ, USA). PCR Genotyping was then performed using Phire Hot Start II DNA Polymerase with the following primers: *5′-Lck*: GTA ACA GTC GGC AGG AGT ATT TAT C and *3′-Lck*: GGA CAG AGC CTC CCT GGT TAT GAA according to the manufacturer instructions.

3.3. Western Blot Analysis

After 24 h of culture in serum-free medium, the cells were treated with human sRANKL (100 ng/mL) for 0, 5, 10, and 15 min at 37 °C. The cells were lysed in radio-immunoprecipitation (RIPA) buffer (150 mmol/L NaCl, 5% Tris, pH 7.4, 1% NP-40, 0.25% Na deoxycholate, 1 mmol/L Na_3VO_4, 0.5 mmol/L PMSF, 10 mg/mL leupeptin, and 10 mg/mL aprotinin) on ice. Lysates were cleared of debris by centrifugation at 10,000 × *g* for 15 min at 4 °C. Total protein concentration was determined with the BCA protein assay kit (Pierce Chemical, Waltham, Ma, USA) according to the manufacturer's instructions. Proteins were run on 10% SDS-PAGE and transferred to Immobilon- P membrane (Millipore, Bedford, MA). The membrane was blotted with antibodies to β-actin (1/1000, A5060, Sigma-Aldrich, Saint-Quentin Fallavier, France), RANK (1/1000, AF683, R&D systems (Abington, UK)), total p38 (1/1000, #9212, CST) and phospho-p38 (Thr180/Tyr182, 1/1000, #9211S, CST). The labeled proteins were detected using Pierce® ECL Western Blotting Substrate (Thermo Scientific) according to the manufacturer's recommendations.

3.4. Zymography Assay

After two days of culture in complete media, the cells were incubated for 24 h in serum-free media, in the presence or absence of sRANKL (100 ng/mL). After this culture period, MMP2 and MMP9 activities were analyzed in cell culture supernatants by zymography in 10 % SDS polyacrylamide gels containing 1 mg/mL gelatin (Sigma-Aldrich). Following electrophoresis, the gels were washed twice in 2.5% Triton X-100 for 30 min and incubated in 50 mM Tris-HCl, and 2 mM $CaCl_2$ for 24 h at 37 °C. The following day, the gels were stained with 0.05% Coomassie brilliant blue (20% isopropanol, 10% acetic acid). Proteinase activity was visible as unstained regions. Gel pictures were taken using a G-box (Syngene, Cambridge, UK).

3.5. Flow Cytometry

The cells were harvested by incubation for 10min with a versene-EDTA solution (Lonza, Walkersville, MD, USA), followed by the addition of complete media to stop the reaction. 2 x 10^5 RANK-overexpressing cells or T-cells were stained with an anti-human RANK/TNFRSF11A

antibody (AF683, R&D Systems) or a PE rat anti-mouse CD3 molecular complex (561799, BD Biosciences) respectively, or a control isotype for 1h at 4 °C. RANK-overexpressing cells were then washed twice and stained with Donkey Anti-Goat IgG (Alexafluor 488) (Ab150129, Abcam, Cambridge, UK) for 30 min at 4 °C. Finally, the cells were washed and re-suspended in 300 μL of PBS and the staining was analyzed with a FC500 flow cytometer (Beckman Coulter, Villepinte, France) using CXP Analysis software version 2.2. (Beckman Coulter). Similarly, T cells were immediately stained after purification by PE rat anti-mouse CD3 molecular complex (561799, BD Biosciences) for 1 h at 4 °C.

© 2018 by the authors. Licensee MDPI, Basel, Switzerland. This article is an open access article distributed under the terms and conditions of the Creative Commons Attribution (CC BY) license (http://creativecommons.org/licenses/by/4.0/).
